# Drug Delivery Systems Based on Metal–Organic Frameworks for Tumor Immunotherapy

**DOI:** 10.3390/pharmaceutics17020225

**Published:** 2025-02-10

**Authors:** Ning Yang, Zongyan He, Tianqun Lang

**Affiliations:** Lin Gang Laboratory, Shanghai 200031, China; yangning@lglab.ac.cn (N.Y.); hezongyan0379@lglab.ac.cn (Z.H.)

**Keywords:** MOFs, tumor immunotherapy, drug delivery

## Abstract

Metal–organic frameworks (MOFs) are a class of inorganic-organic hybrid nanoparticles formed by the coordination of metal ions/clusters and organic ligands. Due to their high porosities, large surface areas, adjustable structures, and responsiveness to light/sound, etc., MOFs have shown great clinical potential in the field of tumor therapy. Tumor immunotherapy exerts antitumor effects through reshaping tumor immune microenvironment, showing significant preclinical and clinical advantages. Based on the mechanisms of immunity activation, the tumor immunotherapy agents can be divided into chemotherapeutic agents, immunomodulators, enzymes, tumor vaccines and oligonucleotide drugs, etc. Herein, we review the MOFs-based drug delivery systems for tumor immunotherapy. The classification of MOFs, followed by their antitumor immunity activation mechanisms, are first introduced. Drug delivery systems based on MOFs with different immunotherapy agents are also summarized, especially the synergetic immunity activation mechanisms triggered by MOFs and their loadings. Furthermore, the merits and drawbacks of MOFs and the potential strategies for MOFs to promote their clinical applications are discussed.

## 1. Introduction

Cancer accounted for nearly 10 million deaths—approximately one-sixth of the total—in 2020, making it a leading cause of death [1]. Tumor immunotherapy exerts antitumor effects by reshaping the tumor immune microenvironment (TIME) to elicit antitumor immune responses and surmounting immune evasion, which has led to a paradigm shift in the treatment of cancers [2,3]. Several immunotherapeutic agents have been used in the clinical management of cancers, conferring significant clinical advantages [4,5].

The tumor microenvironment (TME) is a complex ecosystem, encompassing tumor cells, stromal cells, immune cells, tumor blood vessels, etc. [6]. These jointly foster a hypoxic, acidic, and nutrient-deficient microenvironment that contributes to an immunosuppressive phenomenon known as the “cold” tumor [7]. Immunotherapeutic drugs activate the tumor immunological microenvironment and transform “cold” tumors into “hot” ones, which can be divided as follows: (1) chemotherapeutic agents with immunogenic cell death (ICD) effects; (2) immunomodulators; (3) enzymes; (4) tumor vaccines; and (5) oligonucleotide drugs, among others.

**Figure 1:**
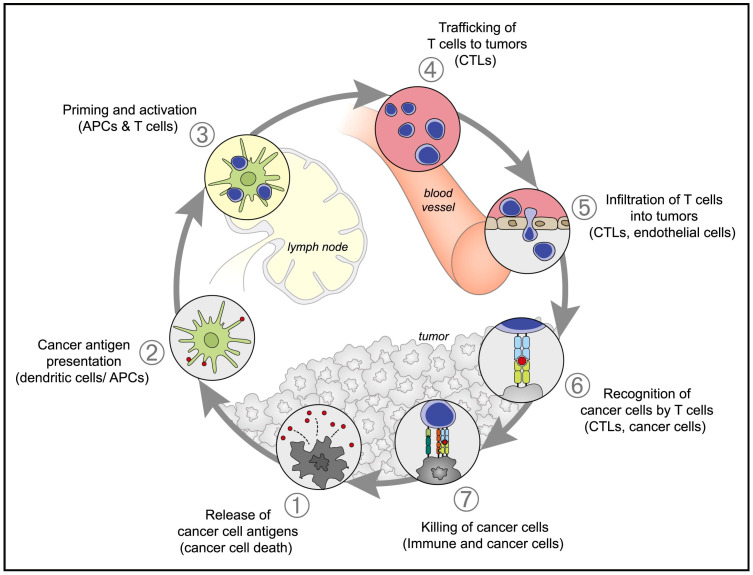
The cancer-immunity cycle. Adapted with permission from ref [8]. Copyright 2013 Elsevier.

Despite different mechanisms, these agents ultimately lead to initiate antitumor immunity through the following steps: (1) the release of cancer cell antigens to trigger the immune response; (2) the recruitment of antigen-presenting cells (APCs), such as macrophages and dendritic cells (DCs); (3) the facilitation of macrophage polarization and DC maturation; (4) migration of DCs to tumor-draining lymph nodes (TDLNs); (5) the infiltration of cytotoxic T cells (CTLs); and (6) the elimination of tumor cells by CTLs (Figure 1) [8]. Nonetheless, under the regulation of various mechanisms, such as diminished antigen presentation, secretion of immunosuppressive factors, recruitment of immunosuppressive cell populations, upregulation of negative regulatory pathways, etc., tumor immunotherapy still faces numerous challenges [9]. In addition, tumor cells evade immunologic surveillance through diverse drug resistance mechanisms, including primary, adaptive, and acquired resistance, thus limiting the effectiveness of immunotherapy [10]. The judicious design and functional development of delivery systems hold promise as a potent method to improve the effects of tumor immunotherapy.

The stable and highly targeted drug delivery systems utilizing nanotechnology improve the effects of cancer immunotherapy. Metal–organic frameworks (MOFs) were first proposed in 1995 [11], which referred to a class of inorganic-organic hybrid nanoparticles formed by the coordination of metal ions/clusters and organic ligands. Typically characterized by regular polygonal rigid structures and high porosity, MOFs exhibit flexible and adjustable structures, making them burgeoning candidates as drug delivery carriers [12,13,14]. MOFs have the following advantages: (1) the high porosity and large surface area provide ample space for drug encapsulation, thereby improving the drug-loading capacity; (2) the wide variety of organic ligands endows MOFs with designed multifunctionality, as many ligands can serve as functional sites for specific molecular recognition; (3) the structure of MOFs can be flexibly adjusted to cater to different active ingredients; (4) the surface of MOFs can be modified for improved biocompatibility, biodegradability and the capability of responsive drug release. First used in biomedical imaging research in 2006 [15], MOFs have shown great clinical potential in the field of tumor therapy, with NCT 03444714 marking MOFs to enter clinical trials as a radiosensitizer for tumors [16]. Studies employing MOFs as delivery carriers for tumor immunotherapy are emerging, providing important references for MOFs in clinical cancer immunotherapy transformation.

**Figure 2:**
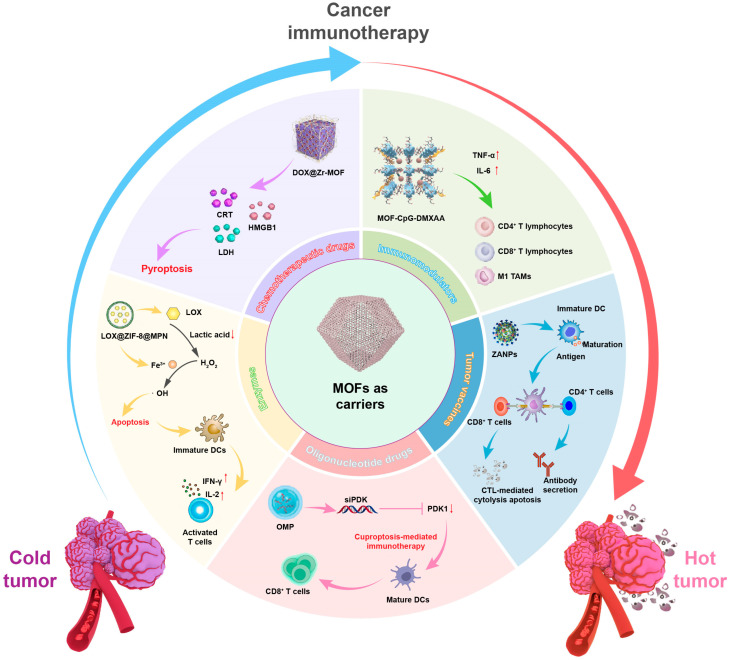
Metal–organic frameworks as carriers for cancer immunotherapy.

Recent studies have shown that the delivery of tumor immunotherapy drugs by MOFs can elicit a synergistic activation of tumor immunity through multiple pathways, thus improving the therapeutic effects. Herein, we summarized the relevant studies, most of which are within the last five years, on tumor immunotherapy using MOFs-based delivery systems, encompassing studies on the tumor immunostimulatory effects and mechanism of MOFs alone and MOFs as a delivery carrier of chemotherapy drugs, immunomodulators, tumor vaccines, and oligonucleotide drugs (Figure 2). In this review, immunotherapeutic agents encapsulated within MOFs were employed as the classification criteria, offering an innovative perspective of MOFs. This provided a new viewpoint and facilitated researchers to quickly navigating their interested domain. After that, we discussed the prospects of MOFs in tumor immunotherapy.

## 2. Classification and Drug Loading of MOFs

**Figure 3:**
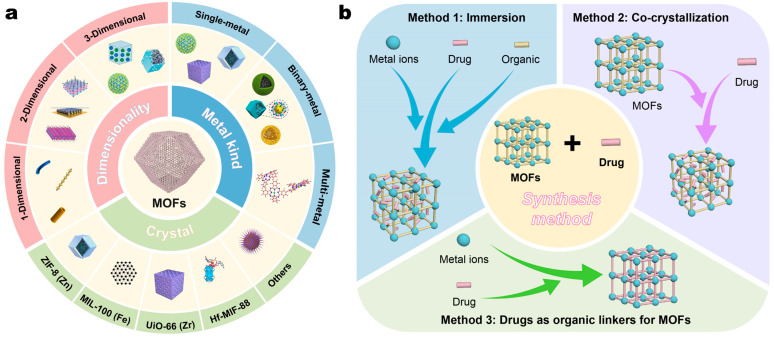
(**a**) The classification and of MOFs. (**b**) The drug-loading methods of MOFs.

The synthesis of MOFs is based on the coordination bond between metal ions/clusters and organic ligands, therefore combining the merits of organic substances and inorganic molecules. Owing to the extensive variety of metal ions/clusters and organic ligands, the structures of MOFs are theoretically infinite [17]. To date, over 80,000 types of MOFs have been designed and synthesized, showcasing a wide application prospect. According to the kind amount of metal ions/clusters, MOFs can be categorized into single-metal MOFs, binary-metal MOFs, and multi-metal MOFs. Based on the type of metal ions/clusters, prominent research focus includes the ZIF-8 based on zinc (Zn) and imidazole ligands, MIL-100 synthesized by iron (Fe) and carboxylate ligands, and UiO-66 formed by zirconium (Zr) and terephthalic acid ligands, etc. Dimensionally, MOFs can be classified into one-dimensional rod-shaped MOFs (1-D MOF rods), two-dimensional sheet-like MOFs (2-D MOF sheets), and three-dimensional nanoparticles MOFs (3-D MOF NPs) (Figure 3a). The synthetic methods for MOFs include self-assembly, solvothermal, electrochemical, mechanical grinding, microwave, ultrasonic techniques, etc. [18]. The solvothermal method, which operates under high temperature and pressure conditions, yields MOFs of enhanced uniformity. Microwave synthesis is rapid and efficient, and self-assembly methods with its mild conditions and ease of operation have emerged as a preferred technique in recent years.

MOFs exhibit profound advantages as drug carriers. During the synthesis of MOFs, the metal ions/clusters as centers are interconnected via coordination and covalent bonds, constructing a framework that boasts a remarkably high specific surface area and a porosity of up to 90% [17], thereby providing an enormous space for drug loading. For example, the MIL-100 and MIL-101 MOFs constructed by Horcajada et al. accommodated ibuprofen at a loading capacity of up to 60% [19]. The surface of MOFs can be modified to adjust their properties, such as enhancing stability, endowing targeting ability, altering surface charge, improving water solubility, and reducing cytotoxicity, thus further improving the performance of MOFs as drug carriers. For example, the chemical and colloidal stabilities of MIL-100 NPs were improved with polyethylene glycol (PEG) surface modification [20], and ZIF-8 NPs were endowed with tumor targeting ability with methoxy-PEG-folate (PEG-FA) coating [21]. Another instance involved modifying the surface of MOFs with hyaluronic acid (HA) to endow them with responsiveness to hyaluronidase in the TME [22]. Moreover, due to the presence of metal ions, MOFs are responsive to stimuli such as sound, light, magnetic fields, and pH, and this feature can be further enhanced by incorporating materials like sonosensitizers and photosensitizers. The advantages of MOFs as drug carriers have made them shine in antitumor therapy and also possess great potential in tumor immunotherapy.

MOFs can achieve drug-loading through several methodologies (Figure 3b). These are as follows. (1) Immersion: the drug molecules enter the pores of MOFs or adhere to their surfaces through host–guest interactions or electrostatic adsorption by simply blending a drug solution with MOFs [23]. (2) Co-crystallization, also known as the “one-pot method: the drug molecules are directly added into the synthetic solution containing metal ions and organic ligands during the synthesis of MOFs, where the drug co-crystallizes with MOFs, forming the MOF skeletons with the drug molecules inside [24,25]. (3) Drug as organic ligands: the drug molecules can work as organic ligands to form MOFs [26], and this method obviates the addition of organic ligands and significantly improves the drug-loading capacity.

In the meantime, the problems MOFs are facing as drug carriers should not be ignored. Among these issues, toxicity and biocompatibility are the first two to be solved. The toxicity of MOFs is deeply dependent on the concentration [27], and both the metal and organic linkers have effects on it. For example, MOFs containing Fe show less cytotoxicity than those containing Zr or Zn [28,29]. Furthermore, the hydrophobic–hydrophilic balance is the main factor to influence the toxicity of MOFs, since it has a direct effects on the removal speed of the nanoparticles. Moreover, the cellular uptake speed of MOFs is related to their cytotoxicity [28]. To better improve their biocompatibility and reduce their toxicity, surface modification is generally employed on MOFs. PEG [20], folic acid [30], and hyaluronic acid [31] are common surface modifications for MOFs, which also have surface functionalization effects. Another important concern comes to their stability, which has a deep impact on the distribution, biodegradability and metabolism in vivo. Most MOFs are stable to heat, since they can be synthesized under high temperature conditions. Zn-based MOFs are easily degraded under acidic conditions, endowing them with the TME responsive characteristics [21]. MOFs of MIL family degrade quickly in PBS or cell culture media due to the presence of phosphate ions, while NU-1000 degraded slowly under high concentrations of them [32]. As relative new carriers for drugs, there are limited studies on MOFs’ release dynamics and metabolism, especially in vivo ones [33].

## 3. MOFs as Drug Delivery Carriers for Tumor Immunotherapy

### 3.1. Tumor Immunity Activation Effect of MOFs

**Figure 4:**
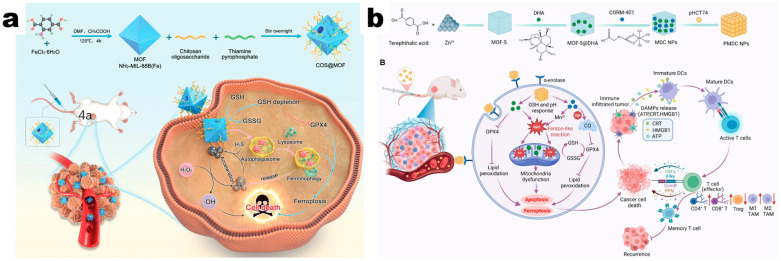
(**a**) Schematic diagram of COS@MOF with a nano-enzyme effect upon internalization by colorectal cancer cells. Reproduced with permission [34]. Copyright 2023, Wiley-VCH GmbH. (**b**) Schematic illustration of the synthesis process of PMDC NPs and their effects on tumor immunotherapy. Reproduced with permission [45]. Copyright 2024, Elsevier B.V.

As a kind of potential carrier material for antitumor drugs, MOFs’ antitumor immunity activation ability has been progressively found by researchers. Notably, the induction of ferroptosis by iron ions is a mechanism which has been well-recognized. For instance, Du et al. developed a delivery system, denoted as COS@MOF, where MIL-88B(Fe) was etched by thiamine pyrophosphate (TPP) and modified with oligo-chitosan. This system exerted a nano-enzyme effect upon internalization by colorectal cancer cells [34]. This led to the depletion of glutathione (GSH), thus inhibiting the activity of glutathione peroxidase 4 (GPX4) and inducing ferroptosis. Concurrently, Fe^2+^ released by COS@MOF accelerated the conversion of H_2_O_2_ into hydroxyl radicals, thereby contributing to the cytotoxicity against tumor cells (Figure 4a). Nevertheless, the immune activation effects of this mechanism warrant further investigation. The study conducted by Ploetz et al. had corroborated this deduction [35]. They conducted a drug delivery system named Lip-MOF by MIL-100(Fe), coated with DOPC. Upon cellular uptake, the released Fe^2+^ not only induced apoptosis in tumor cells but also activated the caspases-dependent pathway, causing the release of gasdermin (GSDM) and the production of IL-1β. The Fe^2+^ also initiated an inflammatory form of programmed cell death and subsequently stimulated the immune system to mount an antitumor response. The immunostimulatory effects of Fe-MOFs have aroused extensive interest. Inspired by this, whether MOFs composed of other metals, such as commonly utilized Mn-MOFs, Zn-MOFs, and Cu-MOFs, exhibit analogous antitumor immune responses has attracted researchers’ interest. Liu et al. conducted a drug delivery system, denoted as Mn-MOF@PEG, by modifying Mn-MOFs with PEG for pancreatic cancer [36]. The findings indicated that Mn^2+^ could increase the percentage of DCs and promote their maturation in the TME, thus enhancing the tumor immune profile. Ding et al. [37] explored the immunity activation effects of Zn-MOFs, specifically ZIF-8 nanoparticles (NPs), and found that ZIF-8 NPs responsively released Zn^2+^, inducing a sudden surge in ionic concentration and intracellular osmotic pressure. As a result, the cysteine-aspartate-specific protease-1 (Caspase-1)/gasdermin D (GSDMD)-dependent pyroptotic pathway was activated to achieve antitumor immune response.

The tumor immunity activation of monometallic MOFs has been widely validated; therefore, the inclusion of multiple metal types in MOFs may elicit synergistic antitumor immune responses. Dai et al. [38] constructed bimetallic MOFs, named Gd-MOF-5, which could release both Gd^3+^ and Zn^2+^ in murine breast cancer 4T1 cells. Gd^3+^ competed with Ca^2+^ to bind TMEM 16F, a Ca^2+^-gated ion channel, leading to its inactivation and the inhibition of phosphatidylserine (PS) externalization. Additionally, the overload of Zn^2+^ induced mitochondrial dysfunction, activated endoplasmic reticulum (ER) stress, and disrupted cellular calcium homeostasis, finally leading to ICD. These two types of metal ions individually induced tumor immunity activation via two distinct pathways, resulting in significantly enhanced outcomes in cancer immunotherapy. Yan et al. [39] further developed trimetallic MOFs composed of Cu, Au, and Zn, and loaded with photosensitizer purpurin 18 (P18), to fabricate an immunostimulatory nanoreactors denoted as ACS-Z-P NPs. Upon laser irradiation, a sudden release of Zn^2+^ induced Caspase 1/GSDMD-dependent pyroptosis and ICD. Concurrently, the active site Au-Cu2-xSe facilitated the production of reactive oxygen species (ROS) through a Cu-mediated Fenton reaction, thereby triggering the activation of inflammatory cells. As a result, damage-associated molecular patterns (DAMPs) were released and, in turn, synergistic immunity activation effects were initiated, leading to the reshape of TIEM.

In summary, the antitumor immunity induced by MOFs is primarily contingent upon the metal ions they contain. On the one hand, the degradation of MOFs can release metal ions, leading to a sudden increase in intracellular osmotic pressure, thereby activating the pyroptosis pathway. On the other hand, MOFs catalyze the generation of ROS with their functions as nanozymes, inducing cell pyroptosis, and activating antitumor immunity. The antitumor immunogenicity of MOFs augments their advantage as antitumor drug carriers, and the subsequent loading of various immunostimulatory agents holds the promise of a synergistic effect greater than the sum of parts, delivering better tumor immunotherapy outcomes.

### 3.2. MOFs as Carriers of Chemotherapeutic Drugs for Cancer Immunotherapy

Although chemotherapy is a paramount therapeutic modality for clinical tumor treatment, its immunosuppressive side effects significantly diminish its efficacy. Recent studies have indicated that certain chemotherapeutic drugs, such as doxorubicin (DOX) [40], oxaliplatin [41], and paclitaxel (PTX) [42], can active tumor immunity by inducing immunogenic cell death. The pro-apoptotic effects of chemotherapy drugs combined with continuous tumor immunity activation would synergistically achieve continuous killing of tumor cells. Since MOFs can effectively activate antitumor immunity, utilizing MOFs as carriers of chemotherapeutic agents will achieve dual effects of drug delivery and immunity activation. The expansive drug-loading capacity and unique pH responsiveness of MOFs render them ideal carriers for chemotherapeutic drug to achieve enhanced tumor immunotherapy effects. As a representative, Fe-MOFs loaded with chemotherapeutic agents effectively achieve synergistic efficacy of chemotherapy and ferroptosis. Moreover, cunningly selected organic ligands (such as the carbonic anhydrase IX (CA IX) inhibitor—fumarate [43]) or combinations with other metals (such as Mn [44]) can promote ferroptosis or amplify immune effects, yielding superior antitumor efficacy. In addition to relying on Fe-MOFs, MOFs based on other metal can also activate ferroptosis pathways by loading with corresponding chemotherapeutic agents. For example, Yang et al. employed Zn-MOFs loaded with dihydroartemisinin (DHA) and CORM-401 for the treatment of colorectal cancer [45]. Both DHA and CORM-401 induced ferroptosis and elevated ROS levels to promote apoptosis, thereby releasing tumor-associated antigens (TAAs) and DAMPs to promote DC maturation and activation of CLTs, and finally achieved sustained and potent antitumor effects (Figure 4b).

Caspase-3 is one of the key proteins in the cell pyroptotic pathway, and the activation of it elicits the upregulation of the tumor suppressor gene gasdermin E (GSDME) and activates the pyroptosis switch in cancer cells. This process leads to the formation of GSDME-N aggregates, perforating the cell membrane, and finally resulting in cell swelling, lysis, and death, with the concomitant release of inflammatory cytokines that activate antitumor immunity [46]. Certain chemotherapeutic agents can counteract the immunosuppressive effects of chemotherapy by activating the Caspase-3/GSDME pathway and achieve improved therapeutic efficacy. For example, Zhou et al. [47] constructed a nanotherapeutic system, denoted as (M+H)@ZIF/HA, by loading the chemotherapeutic drug mitoxantrone (MIT) and the DNA-demethylating agent hydrazine (HYD) into Zn-MOFs and modifying it with HA. HYD and MIT effectively activated the Caspase-3/GSDME-signaling pathway and initiated the pyroptotic program, inhibiting T cell paralysis mediated by myeloid-derived suppressor cells (MDSCs). As a result, the TME transformed from “cold” to “hot”, and the tumor was converted into an antigen repository, thereby leading to a robust antitumor immune response and significant tumors elimination in a breast cancer mouse model. Similarly, Wu et al. [48] prepared a tumor-targeted delivery system, MTX-PEG@TPL@ZIF-8, by loading the chemotherapeutic drug triptolide (TPL) into Zn-MOFs and encapsulating methotrexate-polyethylene glycol conjugates (MTX-PEG). MTX facilitated tumor targeting and accumulation, and Zn-MOFs achieved the pH-responsive release of TPL and MTX in triple-negative breast cancer (TNBC). The release of TPL and MTX could promote cell death via cleaved Caspase-9 and cleaved Caspase-3 upregulating. Apart from loading with agents that activate the Caspase-3 pathway, certain MOFs also active Caspase-3 directly by releasing metal ions. For example, Wang et al. loaded DOX into Zr-MOFs for cancer treatment, and results showed that DOX and Zr^4+^ synergically activated the Caspase-3/GSDME signaling pathway and induced rapid pyroptosis [49]. This system presented superb systemic antitumor immune effect when combined with PD-1 checkpoint blockade.

In addition, MOFs can response to light or ultrasound stimuli. MOFs loaded with chemotherapy drugs combined with light or ultrasound elicit potent antitumor immune response. Inspired by this paradigm, Xing et al. [50] developed a micro-robot, denoted as IDN@MC, by loading decitabine, a chemotherapeutic agent, into ZIF-8. The drug-loaded NPs were subsequently phagocytosed by macrophages. Laser induced rapid and long-lasting antitumor immune response via Caspase-3-dependent GSDME-associated pyroptosis, thus modulating the tumor immunosuppressive microenvironment. Similarly, Yang et al. [51] employed Ti-MOFs to encapsulate cisplatin. Combined with sonodynamic stimulation, this delivery system could effectively active antitumor immune and transform the TIME from “cold” to “hot”, thereby suppressing primary tumors and metastases formation.

### 3.3. MOFs as Carriers of Immunomodulator for Tumor Immunity Activation

TME is in an immunosuppressive condition, and the employment of immunomodulators is a direct approach to activating the immune system, which will elicit a more immediate and potent antitumor immune response, thereby prompting the effective elimination of cancer cells. MOFs as carriers of immunomodulators tend to achieve better tumor immunotherapy effects.

#### 3.3.1. MOFs as Carriers of Innate Immune Molecular Receptor Agonists

As an integral component of the immune system, the innate immune pathway is widely present in most cells, which can provide significant potential for enhancing the TIME [52,53]. The heterogeneity of the innate immune pathway in the TME, associated with development stage, pathway status, and specific cell types, holds promise for targeting innate immunity in tumor immunotherapy. Notably, the toll-like receptor (TLR) pathway and the cyclic GMP–AMP synthase (cGAS)-stimulator of interferon genes (STING) pathway are prominent in the antitumor domain.

**Figure 5:**
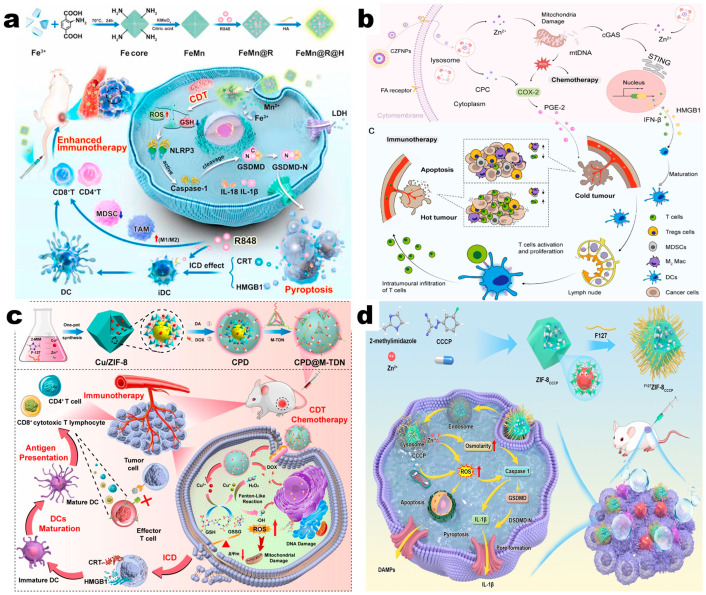
(**a**) Schematic illustration of synthesis tactics and molecular mechanism of FeMn@R@H against tumors. Reproduced with permission [54]. Copyright 2023, Elsevier B.V. (**b**) Harnessing stimulator of interferon genes (STING) and cyclooxygenase-2 (COX-2) signal by steric-hindrance effect tuned nanoreactors for cancer chemoimmunotherapy. Reproduced with permission [61]. Copyright 2024, Elsevier Ltd. (**c**) Schematic illustration showing the preparation process and therapeutic mechanism of CPD@M-TDN. Reproduced with permission [67]. Copyright 2024, Elsevier B.V. (**d**) Schematic illustration of the fabrication and mechanism to induce pyroptosis of ^F127^ZIF-8_CCCP_ nanoparticles for cancer immunotherapy. Reproduced with permission [37]. Copyright 2023, Wiley-VCH GmbH.

TLRs are widely expressed in the spectrum of immune cells, and their activation leads to MyD88- or TRIF-dependent signaling pathways, which further activate NF-κB and innate and adaptive immune responses, improving the efficacy of tumor immunotherapy. Drug delivery systems with MOFs as drug carriers and TLR agonists as active pharmaceutical ingredients (API) can combine the immunity activation effects of both TLR pathway and MOFs, showcasing synergistic efficacy. For example, Feng et al. [54] developed a tumor-targeted drug delivery system, called FeMn@R@H, by using Fe/Mn-MOFs as carriers and TLR agonist resiquimod (R848) as API, and modified the NPs with HA. R848 activated TLR 7/8, triggering downstream pathways, and the substantial release of Fe^3+^ and Mn^2+^ increased ROS and reduced GSH, inducing cell pyroptosis. As a result, FeMn@R@H could promote DC maturation and facilitate the conversion of macrophages from M2 to M1 phenotype, effectively reversing the suppressive TIME and achieving enhanced tumor immunotherapy effects (Figure 5a). Similarly, Pang et al. [55] fabricated drug delivery systems, denoted as isMOFs, which were Zr-MOFs carried with the TLR 9 agonist single-stranded DNA CpG. Subsequently, isMOFs were modified with antiresorptive bisphosphonate, zoledronic acid (ZOL) called BT-isMOFs on the surface. The release of CpG activated TLR 9 and exerted a strong immunostimulatory effect, finally promoting macrophage conversion from M2 to M1 phenotype, thus effectively ameliorating breast cancer-associated osteolysis.

The cGAS-STING-signaling pathway is another crucial immunostimulatory pathway of the innate immune system. cGAS is located in the cytoplasm. Upon activation by double-stranded DNA (dsDNA), cGAS promotes cGAMP synthesis, thereby activating the STING dimer on the endoplasmic reticulum and initiating downstream pathways to express pro-inflammatory cytokines [56]. The cGAS-STING pathway has been implicated in various stages of the cancer immune cycle, such as inducing cancer cell death, enhancing antigen processing and presentation, promoting T cell activation and tumor infiltration, and facilitating T cell recognition and clearance of cancer cells. Delivery systems based on MOFs can achieve tumor-targeted STING agonists delivery, showing excellent antitumor immune effects. Luo et al. [57] loaded the STING agonist 2,3-cyclic GMP-adenosine monophosphate (GA) into Hf-MOFs, constructing a GA-MOF delivery system. GA-MOFs exhibited potent and sustained STING activation effects and could transform tumors into immune hotspots in conjunction with the immune checkpoint inhibitor αPD-L1, achieving excellent systemic immunity activation and distant tumor suppression effects. In addition to the employment of STING agonists, the activation of cGAS/STING pathways induced by other pathways can provide synergistically enhanced effects. Xu et al. [58] developed a delivery system denoted as S@Cu-MOF/PPI by loading polyphenol I (PPI) into spiky Cu-MOFs. The release of Cu^2+^ induced cuproptosis, and PPI triggered cell apoptosis and mitochondrial damage, and subsequently activated the cGAS/STING pathway. These influences synergistically reversed the TIME and significantly eliminated primary tumors and inhibiting the growth of distant tumors.

Furthermore, the concurrent activation of the TLR pathway and STING pathway, as demonstrated by Chen et al. [59], also achieved good tumor immunotherapy effect. They prepared a delivery system named MOF-CpG-DMXAA by using Zr-MOFs as drug carriers and CpG and 5,6-dimethylxanthenone-4-acetic acid (DMXAA) as APIs. DMXAA acted as both a STING agonistic and angiogenic inhibitor. CpG activated TLR4 and downstream pathways, and DMXAA inhibited angiogenesis, achieving starvation therapy, and activated STING pathway. Together, they achieved the effects of DC maturation and tumor-associated macrophages (TAMs) reprogramming, and triggered the systemic immunity activation, thus stimulating a potent antitumor immune response.

#### 3.3.2. MOFs as Vectors for Kinase Inhibitors

Kinases mediate phosphorylation of proteins, thus play a pivotal “switch” role in the whole physiological processes of cells, such as proliferation, apoptosis, migration, and inflammation. The translocation or mutation of kinases may induce the occurrence of cancers, positioning them as potential drug targets. Certain kinase inhibitors can effectively induce immunity activation, thereby exerting tumoricidal effects. The employment of MOFs as vectors of kinase inhibitors in tumor immunotherapy has emerged as a popular research focus [60].

Zhang et al. [61] developed a delivery system, CZFNP, by loading the cyclooxygenase-2 (COX-2) inhibitor C-phycoerythrin (CPC) into ZIF-8 NPs. This system swiftly released Zn^2+^ and CPC in the TME. Zn^2+^ induced mitochondrial damage and mtDNA release to activate the cGAS-STING pathway, and CPC inhibited COX-2 and increased the release of prostaglandin E2 (PGE2). The synergistic therapy activated the immune system and transformed the TIME from “cold” to “hot”, thereby enhancing the therapeutic effects of tumor immunotherapy (Figure 5b). Wang et al. [62] used Gd/Fe-MOFs to deliver the tyrosine kinase inhibitor lenvatinib (LEN) and combined with microwave (MW) hyperthermia for tumor immunotherapy. MW increased the sensitivity of Gd/Fe-MOFs, promoting the production of ROS and ICD. LEN inhibited the activity of fibroblast growth factor receptor 4 (FGFR4) and then facilitated the degradation of programmed death receptor ligand 1 (PD-L1), driving the reprogramming of the TME and activating the immune response to exert tumoricidal effects.

#### 3.3.3. MOFs as Carriers for Immune Checkpoint Inhibitors

Immune checkpoints (ICs) are a series of molecules that regulate the degree of immune activity to prevent excessive activation, playing the role of immune “brake” [63]. However, tumor cells express certain substances to activate immune checkpoints, inhibiting the antigen presentation process and achieving immune evasion. Immune checkpoint therapy (ICT) can remove the suppression effects of tumor cells to immune cells by using immune checkpoint inhibitors (ICIs), ensuring the smooth activation of immune cells and exerting their tumoricidal effects. ICT has offered substantial opportunities and even chances of cure for clinical cancer patients [64].

Currently, several ICIs have been marketed, primarily divided into two categories, which are inhibitors for cytotoxic T-lymphocyte-associated protein 4 (CTLA-4) and those for programmed death receptor 1 (PD-1)/PD-L1. aPD-1 is a commonly used PD-1 model inhibitor, which can directly recognize and bind to PD-1, thereby unblocking immune cell brakes. Cui [65] et al. loaded aPD-1 within Gd- MOF to obtain a Gd/MPC delivery system. This system showed significant activation of T cells and led to the recognition and elimination of tumor cells. Aptamers are a class of RNA or DNA nucleotides that can bind to targets with high affinity. In addition to antibodies, aptamers can also target the PD-1/PD-L1 axis and exhibit the ability to release the immune “brake” [66]. Furthermore, MOF delivery systems with special nanostructure whose convex vertex exerted PD-L1 aptamer effects could also effectively block the immune suppression caused by the PD-1/PD-L1, thus activating the TME immune response [67] (Figure 5c).

Indoleamine 2,3-dioxygenase (IDO) can catalyze the metabolism of tryptophan. The high expression of IDO in tumor cells leads to depletion of tryptophan in the TME, inhibiting the function of tryptophan-sensitive T cells and thereby causing immune evasion. Herein, we may also consider IDO as a type of ICs. MOFs loaded with IDO inhibitor could effectively reduce IDO activity and achieve immune system activation, thus providing significant tumor inhibition effects [68]. Similarly, signal regulatory proteins (SIRPs) are another site for tumor cells to evade immune surveillance, as tumor cells express CD47 to specifically bind to macrophages and prevent their phagocytosis. When α CD47, a CD47 antibody, was loaded into Al-MOFs for cancer treatment, it could bind to CD47 on the surface of cancer cells, relieving CD47 signal-blocking and leading to immunity activation [69].

#### 3.3.4. MOFs as Carriers of Programmed Cell Death Inducers

Programmed cell death (PCD) includes apoptosis, autophagy, and pyroptosis, as well as ferroptosis and cuproptosis, have been identified in recent years. PCD inducers can effectively induce one or several corresponding PCD, exerting antitumor effects. Metal ions released from MOFs have the potential to induce PCD; therefore, MOFs- as PCD-inducer carriers further amplify the PCD effects to achieve a synergistic antitumor immune activation.

Pyroptosis is a kind of lysate and inflammatory programmed cell death pathway different from apoptosis, which induces a strong inflammatory response and contribute to tumor regression [70]. The inherent capacity of MOFs to induce pyroptosis will amplify the pyroptotic signaling when combined with pyroptosis inducers, contributing to the stimulation of antitumor immunity and the enhancement of antitumor efficacy. This was confirmed by the study of Ding et al. [37], where a delivery system, denoted as F127ZIF-8CCCP, was developed by loading cyanoacetate m-chlorophenylhydrazone (CCCP) into ZIF-8 and modifying their surface with Pluronic F127. Zn^2+^ worked as the pyroptotic inducer by activating the Caspase-1/GSDMD pathway, and CCCP elevated ROS levels and magnified the pyroptotic signal, finally leading to synergistic anticancer immunotherapy (Figure 5d). Cuproptosis is a novel mode of cell death caused by excess intracellular coppers, and it mainly functions through the degradation of Fe-S cluster proteins and proteotoxic responses by stimulating the sulfur-acylation aggregation process of mitochondria-related proteins [71]. Similarly to the above study, Cu-MOFs successfully induced cuproptosis, and the cuproptosis-inducer elisomol (ES) augmented this effect, leading to strong ICD and reshaping the TIME. As a result, this Cu-MOFs based-delivery system effectively inhibited the growth of breast tumors [72].

### 3.4. MOFs as Carriers of Enzyme for TIME Reversal

The tumor microenvironment is characterized by features such as hypoxia, acidity, and elevated ROS levels, which play a key role in tumor immunosuppression. Some of these features can inhibit the activity of immune cells and facilitate tumor immune evasion. For example, a relatively low pH may induce cell acidosis and lead to the hematogenous and lymphatic spread of tumor cells, thus worsening the long-term prognosis of patients [73].

The reduced pH in the TME is primarily due to lactate (LA) accumulation. As a product of tumor cell metabolism, LA has been recently identified to act as an “accomplice” of tumor. LA binds to Alanyl-tRNA synthetase 1 (AARS1) to catalyze the formation of LA-AMP complex that promotes the lactylation of the p53 protein, leading to its dysregulation. This, in turn, hampers cell cycle regulation and stimulates tumor growth, thereby facilitating the invasiveness, metastasis, angiogenesis, and immune evasion of cancer cells [74,75]. The introduction of lactate-metabolizing enzymes into the TME can effectively reduces LA levels, presenting a promising strategy for antitumor immunity activation. This was confirmed by the study of Zhou et al. [76]. Lactate oxidase (LOX) loaded within Zn-MOFs depleted the LA levels in the TME and restored vascular normalization, promoting the infiltration of CTL. In another study, LOX was co-delivered with small interfering RNA (siRNA) targeted against monocarboxylate transporter 4 (MCT4) by Fe-MOFs [77]. LOX catalyzed the metabolism and siRNA inhibited the efflux of LA, leading to a synergetic LA-reduced and activated immune system.

**Figure 6:**
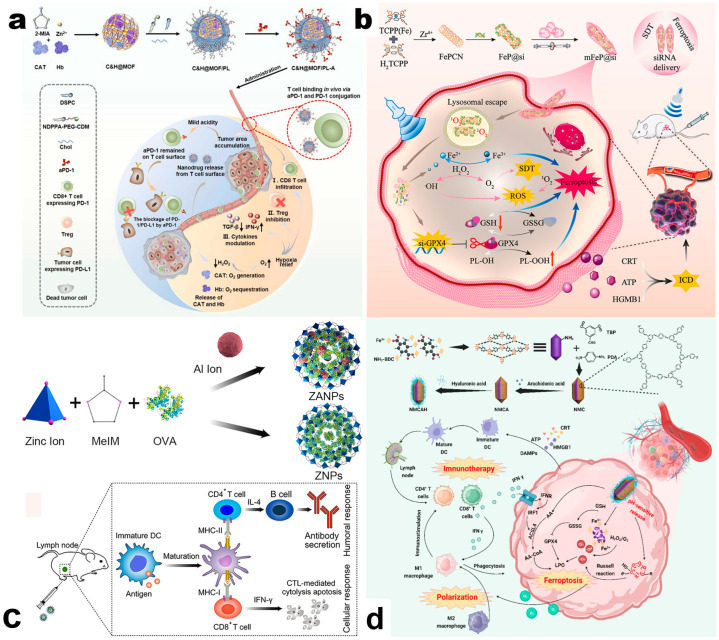
(**a**) Illustration of the synthesis of nanomedicine and the therapeutic mechanism. Reproduced with permission [83]. Copyright 2024, Elsevier Ltd. (**b**) Schematic of the preparation and therapeutic mechanism of mFeP@si. Reproduced with permission [88]. Copyright 2024, Mater. Elsevier Ltd. (**c**) Schematic illustration of the synthetic and antitumor immune potency of aluminum-integrated antigen-MOF (ZANPs). Reproduced with permission [96]. Copyright 2019, Elsevier B.V. (**d**) Schematic illustration of the synthetic route and therapeutic mechanism of NMCAH. Reproduced with permission [99]. Copyright 2024, Elsevier Ltd.

Another major feature of the TME is the increased GSH. As a critical regulator of tumorigenesis, progression, and metastasis, GSH aids in the elimination of ROS and reduces the sensitivity of tumor cells to oxidative stress [78,79]. The hypoxia phenomenon is also worthy of attention. Hypoxic conditions activate the hypoxia-inducible factor 1α (HIF-1α) which enables tumor cells to survive at lower oxygen levels. As a result, the proliferation, invasion, and metastatic capabilities of cancer cells are improved and the immunosuppression of the TME is enhanced [80]. Catalase (CAT) positively influences both factors by reducing GSH levels and alleviating hypoxia [81]. When combined with MOFs, its immune-boosting effect was further reinforced. The drug system based on Cu-MOFs loaded with CAT triggered cuproptosis to induce ICD, depleted GSH, and promoted H_2_O_2_ decomposition into O_2_ to relieve hypoxia, effectively reversing TIME suppression [82]. CAT loaded in Zn-MOFs showed similar antitumor immunotherapy effects [83] (Figure 6a).

Apart from the enzymes aiming for the balance of the TME, other enzymes can also stimulate antitumor immune response through certain catalytic actions on intracellular substances. For example, Yang et al. [84] delivered glucose oxidase (GOx) by cancer cell membrane-coated Fe-MOFs for cancer treatment. GOx catalyzed glucose to produce H_2_O_2_, leading to ROS generation and subsequently inducing ICD.

### 3.5. MOFs as Carriers of Oligonucleotide Drugs to Overcome Immunotherapy Tolerance

Oligonucleotide drugs, also known as small nucleic acid drugs, include siRNA, microRNA (miRNA), and antisense oligonucleotides (ASO), among others. Oligonucleotide drugs achieve therapeutic effects through specifically silencing the expression of disease genes; therefore, they have the capacity to develop agents for untargetable or undruggable diseases [85]. Specifically designed siRNA or miRNA can downregulate the expression of proteins. while circular DNA drugs can introduce target genes into specific cells to elevate protein levels, thereby exerting effects on targets. The silencing or activation effects of immune responses contribute to antitumor efficacy. MOFs, with positively charged surfaces and expansive internal cavities, serve as ideal carriers for nucleic acid drugs, protecting them from enzymatic hydrolysis, increasing their loading, and promoting lysosomal escape [86].

siRNA, composed of 21–23 nucleotide-long double-stranded RNA molecules, can integrate into the RNA-induced silencing complex (RISC) and lead to the degradation of the passenger strand (sense strand), thus inducing the subsequent post-transcriptional gene silence and effectively reducing target protein levels [87]. As introduced above, Cu-MOFs have the ability to trigger cuproptosis [58,72]. Similarly, Fe-MOFs can induce ferroptosis. Sun et al. employed Fe/Zr-MOFs to load siRNA-targeting glutathione peroxidase 4 (GPX 4), constructing a biomimetic nanomedicine delivery system named mFeP@si [88]. The release of Fe^2+^ increased ROS levels and led to ferroptosis. siGPX4 silenced GPX 4 expression and caused the accumulation of toxic phospholipid hydroperoxides (PL-OOH), thus amplifying the ROS storm. As a result, this delivery system induced robust ICD and reversed the immunosuppressive TME, enhancing antitumor immune effects (Figure 6b). In another study, siRNA targeting ataxia-telangiectasia and rad3-related (ATR) and the chemotherapeutic drug DOX were co-loaded in ZIF-8 NPs to modulate tumor gene expression profiles. DOX induced DNA damage to tumor cells, and siATR silenced the expression of ATR protein, inhibiting DNA repair. The viability of tumor cells was reduced, and their immunogenicity was increased. As a result, the TME was transformed from suppressive to activated, achieving combined chemotherapy and immune stimulation for pancreatic cancer [89].

miRNA is a type of oligonucleotide drug that consists of 19–25 nucleotide-long double-stranded RNA molecules. Similarly to siRNA, miRNA drugs also aim for the reduction in protein expression levels. The difference is that miRNA can silence the target through RISC or induce mRNA degradation by direct binding [90]. Cui et al. developed a delivery system, denoted as OMV@ZIF@pre-miRNA, by loading miRNA within ZIF-8 and coating with bacterial outer membrane vesicles (OMV-PD-1) that specifically express PD-1 [91]. PD-1, on the surface, mediated precise tumor targeting and triggered the immune checkpoint inhibition to activate antitumor immunity. miR-34a inhibited the translation of the target mRNA, causing cell cycle arrest and effectively eliminating tumors.

In addition, traditional plasmids and circular DNA also play a significant role in antitumor immunotherapy by introducing the desired genes. In another research, Zhao et al. constructed a gene delivery system using Fe-MOFs MIL-88A loaded with minicircle DNA [92]. The minicircle DNA stably expressed bispecific T cell engager (BiTE) anti-CD3/anti-EpCAM, thus enhancing tumor localization and CTLs recognition, significantly inhibiting tumor growth.

### 3.6. MOFs as Carriers of Tumor Vaccines for Immune Stimulation

Tumor vaccines have emerged as a pivotal strategy in tumor immunotherapy. Tumor vaccines achieve suppression of tumor progression via releasing antigen to stimulate the immune system and eliminate tumor cells. As clinical trials advance, the efficacy of tumor vaccines is increasingly being validated [93]. For a tumor vaccine to exert potent antitumor immunity activation, it is necessary to complete the process of tumor antigen uptake and presentation by DCs, CTLs activation, and the subsequent recognition and destruction of tumor cells by CTLs. However, the immune evasion mechanisms in the TME, such as immune checkpoint inhibition and antigen presentation restriction, significantly impede the efficacy of tumor vaccines [94]. Effectively achieving antigen presentation has become the key to tumor vaccines.

A more direct solution is to increase the antigen levels targeting and accumulating in the tumor site. Ovotransferrin (OVA) is a commonly used antigen model. Delivery systems based on OVA as a major API and MOFs as carriers have shown significant effects on prompting DC maturation and CTL activation, thus stimulating robust tumor-specific immune responses and leading to significant anticancer effects against both primary and distant tumors [95,96] (Figure 6c). In addition to model antigen vaccines, the whole cell cancer vaccines (WCCVs), which use tumor cells as vaccines, contain a full array of tumor-associated antigens (TAAs), thereby activating more comprehensive and potent antitumor immune responses [97]. Yang et al. built novel WCCVs by forming a ZIF-8 shell on tumor cells [98]. Upon ICD of tumor cells, calreticulin (CRT) was exposed and TAAs were released at relative high levels, inducing robust tumor immunity activation.

Beside the above solutions, increasing the antigen presentation efficacy is an alternative way to improve the immunity activation effects of tumor vaccines. For example, Zhang et al. constructed tumor nanovaccine, denoted as NMCAH, based on Fe-MOFs by loading arachidonic acid (AA) and coating 1,3,5-benzenetricarboxaldehyde (TBP), p-phenylenediamine (PDA), and HA [99]. This nanovaccine could improve the tumor immunogenic by triggering and amplifying the ferroptosis effects and lead to the activation of antitumor immunity and tumor-killing effects (Figure 6d). Liu et al. developed a biomimetic nanovaccine based on Mn/Zr-MOFs encapsulating tumor cell membranes (CM) with Ythdf1-targeted short hairpin RNA (shY1) [100]. The gradual release of TAAs, shY1, and Mn^2+^ synergistically activated the cGAS/STING immune pathway, increasing cross-presentation, thus inducing potent antitumor immune response.

The key to tumor vaccines for immunotherapy is to ensure the activation of the whole process. The employment of MOFs as vectors for tumor vaccines help increase the antigen levels, improve the antigen presentation efficacy, lead to refined vaccine efficiency, and promote DC maturation and CTL activation, and help reshape the TIME.

## 4. Conclusions and Prospects

In summary, MOFs have emerged as outstanding candidates for drug delivery systems in recent years, garnering significant interest in tumor immunotherapy. By combining the high safety of organic carriers with the high drug-loading capacity of inorganic carriers, MOFs have benefits such as high porosity, substantial drug-loading capacity, and adjustable structures, as well as responsiveness to optical, acoustic, and pH stimuli. Since MOFs inherently release metal ions, they have the ability to activate tumor immunity. However, the superior immune activation properties of MOFs act as a “double-edged sword”. Cell cytotoxicity, along with its immunity activation ability, reduces the concentration threshold required for application. In order to improve the biocompatibility of MOFs, researchers have conducted various surface modifications on MOFs, such as PEGylation and cell membrane encapsulation. These methods not only improve their biocompatibility, but also endow them with special functionalities, such as tumor targeting, improved solubility, and increased cellular uptake. These advancements are crucial for promoting the clinical application of MOFs and will likely be a focal point of future research endeavors.

In this review, the classification and advantages of MOFs as delivery vectors are introduced. Subsequently, the research progress of antitumor immunotherapy systems based on MOFs is discussed, in accordance with the immunity activation mechanisms of agents loaded in MOFs. A concise overview of MOF-based delivery systems for antitumor immunotherapy introduced in this review is summarized in Table 1. Based on their ability to activate antitumor immunity, MOFs and their loaded agents have synergistic effects in tumor treatment. Herein, delivery systems based on MOFs have been comprehensively utilized for small-molecule drugs, therapeutic antibodies, and nucleic acid-based therapeutics. Their applications for other novel agents, such as gene editors or CAR-T cells, need pioneering investigation. Hopefully, this review can help provide new insights for future studies on MOFs and help promote their clinical applications. 

## Figures and Tables

**Table 1:** MOF-based drug delivery systems for tumor immunotherapy.

MOF Type	Metal Type	MOF Name	Linker	Cargo	Coating	Immune Activation	Reference
Mono-metal MOFs	Fe	COS@MOF	2-aminoterephthalic acid	-	Chitosan oligosaccharide	Ferroptosis	[34]
		Lip-MOF	Tri-mellitic-anhydride	-	1,2-Dioleoyl-sn-glycero-3-phosphocholine (DOPC)	Pyroptosis	[35]
		p-LDM	1,4-benzenedicarboxylicacid (H_2_BDC)	Doxorubicin (DOX)	DOPC DSPE-PEG2000 Pep-DSPE-PEG2000	Ferroptosis	[43]
		IPI549@MOF/CpG NPs	Tannic acid (TA)	IPI549 CpG	-	TLR9 pathway activation	[55]
		MLSLF	Dithiodiglycolic acid	Lactate oxidase, siMCT4)	Folate-liposomes	GSH depletion,·OH generation	[77]
		Fe(SS)DG MOF	Dithiodiglycolic acid	DOX glucose oxidase (Gox)	Cell membrane	ROS-ferroptosis-glycolysis regulation	[84]
		MOF/MC.BiTE	Trimesic acid	Minicircle DNA	-	T cell activation, BiTE expression	[94]
		NMCAH	NH_2_-BDC	Arachidonic acid	Benzaldehyde Phenylenediamine hyaluronic acid	Ferroptosis	[99]
	Zn	^F127^ZIF-8_CCCP_ NPs	2-methylimidazole (2-MIM)	Carbonyl cyano-m-chlorophenone (CCCP)	F127	Pyroptosis	[37]
		PMDC NPs	Terephthalic acid	Dihydroartemisinin (DHA), CORM-401	pHCT74	Apoptosis, ferroptosis	[45]
		(M + H)@ZIF/HA	2-MIM	Hydralazine (HYD), mitoxantrone (MIT)	Hyaluronic acid (HA)	Pyroptosis, MGO reduction	[47]
		MTX-PEG@TPL@ZIF-8	2-MIM	Triptolide (TPL)	COOH-PEG2000-MTX	Cleaved Caspase-9/3 pathway activation	[48]
		IDN@MC	2-MIM	Decitabine	IR-MC	Pyroptosis	[50]
		CZFNP	2-MIM	-	NH_2_-PEG-FA	STING and PEG-2 pathway activation	[61]
		TZDI	2-MIM	DOX IDO inhibitors	3D-printed scaffolds	ICD, ICB	[68]
		LOX@ZIF-8@MPN	2-MIM	Lactate oxidase	Fe-Metallic polyphenol network	Apoptosis, ICD, Vascular normalization	[76]
		C&H@MOF/PL	2-MIM	Catalase, hemoglobin	Apd-1, DSPC, NDPPA-PEG-CDM, cholesterol	Hypoxia relief	[83]
		MOF_DOX@siATR_	2-MIM	DOX siATR	-	siRNA silence ICD	[89]
		OMV@ZIF-8@pre-miRNA	2-MIM	Pre-miRNA	Bacterial outer membrane vesicles expressing PD-1 (OMV-PD-1)	mRNA degradation	[92]
		Apt-Cell@ZIF-8	2-MIM	Cancer cell	-	CRT explosion, tumor antigen presentation	[98]
	Cu	S@Cu-MOF/PPI	Bis(trichloromethyl) carbonate (BTC)	Polyphyllin I (PPI)	-	Cuproptosis, STING pathway, apoptosis	[58]
		Cu(II)-MOF	BTC	Elesclomol (ES)	Polyethylene glycol, (PEG)	Cuproptosis ICD	[72]
		BCMD	1,3,5-Benzenetricarboxylic acid (H_3_BTC)	Catalase, buthionine-sul foximine	Dodecyl-beta-D-maltoside	ICD, cuproptosis	[82]
	Mn	Mn-MOF@PEG	2,5-dihydroxyterephthalic acid (H_4_DOBDC)	-	Dicyclohexyl carbodiimide (DCC), PEG	DCs muturation	[36]
		DOX@Zr-MOF	5,10,15,20-tetrakis(4-carbonxyphenyl) porphyrin (TCPP)	DOX	DSPE-PEG2000-NH_2_	ICD, Caspase-dependent pathway activation	[49]
		BT-isMOFs	Terephthalic acid (H_2_BDC)	CpG	Zoledronic acid (ZOL)	TLR9 pathway activation	[55]
		MOF-CpG-DMXAA	Fumaric acid	5, 6-dimethylflavono-4-acetic acid (DMXAA), CpG	-	Starvation therapy, TLR9 and STING pathway activation	[59]
		cMn-MOF@CM	Benzoic acid	CpG, bovine serum albumin (BSA)	B16-OVA membrane	Antigen presentation	[95]
	Ti	PMPPO	NH_2_-H_2_BDC, BA	Pt O_2_	-	ICD, apoptosis	[51]
	Hf	DBP-Hf MOFs	H_2_DBP	2,3 cyclic guanosine monophosphate—adenosine monophosphate (GA)	-	cGAS-STING pathway activation	[57]
	Gd	Gd/MPC	Fumaric acid	aPD-1	1-Tetradecanol SCC7-cell membrance	ICB, apoptosis	[65]
	Al	ICG-R848@Al-MOFs	NH_2_-BDC	α-CD47, ICG, R848	-	ICB, TLR pathway activation	[69]
Binary-metal MOFs	Gd Zn	Gd-MOF-5	H_2_BDC		TMEM 16F Deactivation ICD	ICD, TMEM 16F deactivation	[38]
	Mn Fe	MMCH	Oleic acid	Cisplatin	HA	Ferroptosis, ICD	[44]
	Fe Mn	FeMn@R@H	2-aminoterephthalic acid, citric acid	R848	HA	Pyroptosis	[54]
	Fe Gd	LEN@Gd/FeMOF-PEG	Trimesic acid Fumaric acid	Levatinib	Mpeg-SH	ICD	[62]
	Cu Zn	CPD@M-TDN	2-MIM	DA, DOX	M-TDN	ICD, GSH depletion	[67]
	Fe Zr	mFeP@si	H_2_TCPP	siGPX4	OS cell membrane	Ferroptosis, GPX4 silence	[88]
	Zn Al	ZANPs	2-MIM	Ovalbumin (OVA)	-	Antigen presentation	[96]
	Mn Zr	Mn/Zr-MOF-shY1-CM	TCPP	shY1-plasmid	Cell membrane	STING pathway activation, Ythdf 1 silence	[100]
Tri-metal MOFs	Cu Au Zn	ACS-Z-P NPs	2-MIM	Purpurin 18	Purpurin 18	Pyroptosis, ICD	[39]
